# *Canadian Journal of Kidney Health and Disease:* a unique launch of a unique journal

**DOI:** 10.1186/2054-3581-1-1

**Published:** 2014-04-07

**Authors:** Adeera Levin, Catherine M Clase, Manish M Sood, Elizabeth Dicks, Marie-Chantal Fortin, Sunny Hartwig, Rachel Holden, Jean-Philippe Lafrance, Anita Molzahn, Norman Rosenblum, Susan Samuel, Steven Soroka

**Affiliations:** University of British Columbia, Vancouver, BC Canada; McMaster University, Hamilton, ON Canada; University of Ottawa, Ottawa, ON Canada; Memorial University of Newfoundland, St John’s, NL Canada; University of Montreal, Montréal, QC Canada; University of Prince Edward Island, Charlottetown, PE Canada; Queens University, Kingston, ON Canada; University of Alberta, Edmonton, AB Canada; The Hospital for Sick Children, Toronto, ON Canada; University of Calgary, Calgary, AB Canada; Dalhousie University, Halifax, NS Canada

## Abstract

Usually inaugural editorials are written by the Editor-in-Chief to describe the scope and vision for the journal to potential authors and readers. This editorial is written by the Editor-in-Chief, the Deputy Editors and the Associate Editors collaboratively as a clear signal that this is a unique and different journal. We will build this journal on a set of principles which are fundamental to improving the outcomes of patients with kidney disease. To that end, we aim to be supportive, to collaborate, to integrate multiple perspectives and to be open to possibilities.

## Résumé

Habituellement, il revient à l’éditeur en chef de rédiger l’éditorial inaugural décrivant la vision et les champs d’intérêts d’un nouveau journal. Le Journal canadien de la santé et de la maladie rénale a choisi de faire les choses autrement. En effet, cet éditorial est le fruit de la collaboration entre l’éditeur en chef et les éditeurs en chef adjoints. Ce journal s’appuie sur des principes qui seront fondamentaux pour améliorer le sort de patients atteints de maladie rénale. Pour y arriver, nous nous engageons à apporter du support aux auteurs, à collaborer, à intégrer différentes perspectives et être ouverts à des nouvelles possibilités.

## Editorial

This inaugural virtual edition of the *Canadian Journal of Kidney Health and Disease* – the official journal of the Canadian Society of Nephrology launches now following Kidney Month in Canada, as well as World Kidney Day, March 13th 2014, in recognition of our commitment to international perspectives. We have a vision of a high-quality journal publishing basic, translational, clinical, and health-services research in the field of kidney disease, dialysis, and kidney transplantation, with the mandate to promote and advocate for kidney health as it affects Canadians and people worldwide. The Journal has, and will continue to develop, an explicit culture of enquiry, sharing and knowledge dissemination for nephrologists and others involved in the care and prevention of kidney disease in Canada and the world.

All journals strive for quality in their publications. To attract those publications, our journal is distinguished by:

1. **Supportive review.** Our explicit and unique policy is to address our authors as our colleagues, and to aim to improve the work submitted, regardless of our decision to publish. We will be no less scientifically and constructively critical, but we will write to authors as we would wish to be written to ourselves.
2. **Clear review.** Our associate editors will clearly indicate the major obstacles to publication. We expect authors will respond with openness to feedback, integrity, and clarity, so that most publications will require a single round of scientific revision.
3. **Timely review.** We will provide authors with a review within 2 weeks of receiving a manuscript, or notify authors of the delay and the proposed timeline if we cannot.
4. **Easy formatting.** We recognise the irritation and waste of time involved in formatting a paper differently for each journal and we know that many manuscripts are seen by more than one journal before publication. We will not ask authors to waste time on reformatting for our needs before scientific acceptance. We would like a structured abstract, but can work on that with authors before publication. We ask only that authors limit the abstract to 450 words, main text to 3000 words, and use one of the standard numbered citation formats for references.
5. **Timely publication.** Delay in author submission of revisions is one of the major obstacles to timely publication. When authors receive supportive feedback within 2 weeks of submission, we anticipate that it will be cognitively easier for them to respond: immediately in many cases. If additional experimental work or complex re-analyses are necessary, there will be an inevitable lapse of time, but we encourage all authors to respond as soon as they are able. Once accepted, work can be published in PDF within 2 to 3 weeks.
6. **Recognition of limitations.** All scientific work is limited. We challenge authors to explicitly recognise the limitations of their own work without fear that it will reduce the likelihood of publication. We will acknowledge limitations in full text and abstracts, and will work with authors to highlight the importance of their findings without overstating them.
7. **Openness to creativity and innovation.** We would like to hear from you what you would like to read or to write.
8. **Open access publication.** This translates into work being available, in full text, to anyone with internet access worldwide. This is critical to the dissemination of findings to inform kidney care and research internationally, and particularly important for those working in resource-limited environments. Authors retain the copyright to their published content enabling full rights to reproduce and reuse provided correct attribution is given [1].
9. **Dissemination.** We will push monthly summaries of content, with hypertext links to full text, to all members of the Canadian Society of Nephrology and to all registrants at our website. We have an active publicity campaign reaching out through meetings of societies of nephrology, dialysis, hypertension, and transplantation worldwide that will rapidly increase the prominence and readership of the journal. Those who wish can additionally subscribe to RSS feeds that will push each article as published.

The Journal logo, a variation on the tree of knowledge and of life, is symbolic of the ethos of the journal. Through a philosophy of thoughtful and timely reviews of publications, we hope to foster the growth in knowledge, and thus in wellness of patients living with kidney disease.

While many Canadian kidney researchers are well celebrated and have excellent track records in high impact journals, the importance of their work is often not fully appreciated in Canada. This Journal will afford that opportunity. Furthermore, the Journal will help with knowledge translation of research findings by giving Canadians a voice that can be heard clearly by Canadian health care administrators and policy makers – and therefore, lead to meaningful and necessary changes in health care delivery. Regular high-value features or reviews by Canadians and for Canadians also may be powerful tools for impacting change. In addition, Canadian practitioners and administrators may not always have access to information about important and relevant research and programs outside Canada. Thus, we hope that our emphasis on quality in our publications, our timely, thoughtful and collaborative approach to reviewing, and the open-access publication of their work will attract high-quality submissions from the international kidney community.

The editor-in-chief’s commitment to showcasing excellence in Canadian research, recognition of the importance of the voice of Canadian research contextualized within the Canadian health care environment, and determination to impact policy and practice at home with research by and for Canadians, are critical to her role in this inaugural position. Her international roles and relationships through the International Society of Nephrology, positions the journal uniquely to fulfilling a broader mandate and informing health research and care around the world. The deputy editors and associate editors also have diverse perspectives and spheres of influence: they represent adult and paediatric nephrologists, basic and clinical researchers, administrators, and nurses, hail from across Canada. There is equal representation of both genders on the editorial team. Our managing editor, Elizabeth Dicks, is a PhD Nurse Scientist, with an established career in nephrology research.

Thus, as the official journal of the Canadian Society of Nephrology, we start life with a core of Canadian associate editors encompassing junior, middle and senior investigators. Over time, we will include scientists from other countries. The larger Editorial Board is a diverse and international group, including health policy experts, administrators, health economists, and academics from diverse disciplines, and is representative of the general nephrology community. As a unique endeavor, we have also included on this Editorial Board [2], the trainees from the Kidney Research Scientist Core Education and National Training Program (KRESCENT) program so that they gain experience in peer review and editing, supervised by senior scientists. Thus, we provide for a sustainable future for the Journal. We will invite guest editors for special editions to mark key events or discoveries.

The timing is right for a Canadian nephrology journal. Canadian nephrology administrators and researchers are highly prominent internationally and there is an unprecedented sense of internal and external collaboration in the Canadian kidney community presently. Canadian nephrologists chairing global kidney disease guidelines committees, funding of the Canadian Kidney Knowledge Translation and Generation Network by the Canadian Institutes for Health Research (CIHR), and co-funding of the KRESCENT program by CIHR, Canadian Society of Nephrology and the Kidney Foundation of Canada (KFoC) attest to this. We recognize that there is international competition between journals for impact factor and citations. We believe that established Canadian and international investigators, motivated by a sense of congruence with our mandate and ethos, will publish with us. In addition to this motivation, newer investigators will particularly value the constructive, collaborative and timely nature of our reviewing process.

The goal is to develop a journal that complements existing journals by offering something unique. In addition to reports of completed basic, translational, clinical, economic and policy research, you can expect to read about knowledge translation activities, reports on research team development and outputs, descriptions of provincial structures for renal services, progress in clinical trials being conducted in Canada (across the country or as part of the international community) and high-quality work by fellow, resident and student researchers. Articles describing challenges in health care delivery, and perspectives from different provinces or jurisdictions will be of value to the nephrology community. Work on disadvantaged populations (within and outside of Canada) will bring our collective social conscience into focus and make kidney care relevant for all. Clinical practice guidelines, their rationale, methods, and applicability will clearly differentiate what is evidence-based and what is clinical judgment. Our open-access structure means that we can reasonably consider our readership to include other disciplines and specialties and, in particular we wish to include work that is relevant to allied health, primary care, and non-academics worldwide. Special issues will focus on educational features in both adult and pediatric nephrology, as well as basic and clinical science challenges. We will maintain close linkages with the Canadian Society of Nephrology Education Committee, showcasing the output from our national meetings both by established researchers and by trainees.

We will ensure that the Journal truly reflects the needs of the Canadian Society of Nephrology membership, the Canadian health care system and patients and practitioners within it. We will be sensitive to the international applications and implications of our publications. Through striving for excellence in the quality and relevance of published articles we aim to become a leading national and international nephrology journal. We consider it a privilege to be able to disseminate high quality work, to contribute as a community to that work, and ultimately to improve the lives of those living with kidney disease.

To achieve these aims, we solicit submissions and will do our best to provide thoughtful, constructive and timely review. And above all, we solicit your interest and readership as we set out on this journey.
